# Mathematical modeling of heat transfer in tissues with skin tumor during thermotherapy

**DOI:** 10.1371/journal.pone.0298256

**Published:** 2024-05-16

**Authors:** Hany H. Sherief, Mohamed F. Zaky, Mohamed F. Abbas, Samar A. Mahrous

**Affiliations:** 1 Department of Mathematics, Faculty of Science, Alexandria University, Alexandria, Egypt; 2 Institute of Basic and Applied Science, Arab Academy for Science, Technology and Maritime Transport, Alexandria, Egypt; College of Mathematics and Systems Science, Shandong University of Science and Technology, CHINA

## Abstract

The study of thermal therapy to tumors and the response of living cells to this therapy used to treat tumor is very important due to the complexity of heat transfer in biological tissues. In the past few years, there has been a growing interest among clinicians, mathematicians, and engineers regarding the use of computational and mathematical methods to simulate biological systems. Numerous medical proceedings also employ mathematical modeling and engineering techniques as a means to guarantee their safety and evaluate the associated risks effectively. This manuscript provides an analytical solution used for the first time to study the mechanism of biological thermal response during heat therapy on spheroidal skin tumor. The proposed method used a generalized thermoelasticity model with one relaxation time. The influence of relaxation times on the responses of diseased and healthy tissues is studied and interpreted graphically. Also, the impact of different laser irradiance on the thermal profile of the malignant tumor cells over a period of 2 minutes is interpreted graphically. To investigate the transfer of heat within biological tissues during the thermal therapy, the Laplace transform and inverse Laplace transform methods were applied. A comparison of the present generalized thermoelasticity model and different models based on Pennes bioheat transfer PBT shows that our proposed model yields more realistic and accurate predictions. The current model can be used to explain various therapeutic methods.

## 1. Introduction

Cancer is one of the most dangerous diseases that cause millions of deaths every year around the world. Cancer can be treated in several ways, such as surgery, chemotherapy, radiotherapy or thermal therapies [1]. However, these treatments have numerous side effects that let patients feel intolerable pain. Thermotherapy also known as Hyperthermia is the treatment strategy that disregards the usage of chemicals or harmful radiations [2]. Nevertheless, one of the difficulties in thermal treatments lies in the ability to target and eliminate tumor cells specifically, while avoiding harm to the adjacent normal tissues. Tansey and Johnson [3] have reported that temperatures in the range of 40°C to 45°C can be effective in causing the destruction of malignant cells through thermonecrosis in tumor tissue while minimizing damage to the neighboring healthy tissues. To achieve successful clinical treatment, it is crucial to comprehend the temperature propagation within biological tissue, as this understanding plays a vital role in ensuring high-quality outcomes.

Hyperthermia is a very interesting research area in medicine nowadays, allowing less pain and providing shorter recovery time. It is a non-invasive treatment in which diseased tissues are exposed to high temperatures to destroy the cancerous tumor [4–7]. Several researches have investigated the usage of heat transmission into biological cells, over the past four decades [2, 6–16]. It has been found that treating some types of cancer with chemotherapy or radiation when combined with heat therapy is much more effective than by conventional cancer therapies alone [17–20]. The research conducted by Gozal and Djakaria [21] has shown promising results in the treatment of Ewing Sarcoma which is the second most common bone tumor, predominantly affecting individuals in the age range of 10 to 30 years. Through a combination of chemotherapy and thermotherapy, they increased the 5-year survival rate for patients from less than 10% to more than 60%. Heat therapy enhances the flow of blood and oxygen within the malignant tumor, resulting in increased drug absorption by deeper regions without influencing normal cells [22]. However, the excessive heat exposure to the healthy cells may cause their damage. Hence, the success of the therapy depends not only on the technique applied but on the comprehensive examination of the complicated bioheat transmission operation as well and the manner of temperature rise in living tissues through the thermotherapy [23]. Wust et al. [10], Han [12] and Deatsch et al. [24] studied in detail the thermotherapy techniques.

The accurate interpretation of the thermal process between tissues is very crucial for the improvement of medical technology in treating such fatal disease. Thus, modeling the heat transmission in living cells is a way to achieve this end. Numerous mathematicians and physicists have proposed mathematical techniques to study heat transportation concerns in biological cells [25–28]. Several studies of thermotherapy use Fourier’s law of heat conduction. Although this traditional law is widely used, it fails specially in thermal diffusion conditions, where the heat diffusion exhibits non-Fourier behavior such as short-pulse laser irradiation. A similar phenomenon was noticed in substances with non-homogeneous internal structure, which are analogous in heterogeneity to living cells. Traditional uncoupled thermoelasticity ignores mechanical state influence on temperature, using the heat equation. In 1956, Biot [29] introduced coupled thermoelasticity, addressing this issue but retaining infinite heat propagation speed. Lord and Shulman’s 1967 work [30] for isotropic media replaces the Fourier law with the Maxwell-Cattaneo law, resolving the infinite heat diffusion paradox, later extended by Sherief [31] and Dhaliwal and Sherief [32] in 1980, to cover the anisotropic media. This hyperbolic formulation provides a realistic description of heat propagation, improving our understanding of thermal phenomena and their interaction with mechanical responses.

Pennes [26] studied the temperature distribution in the forearm. He used a modification of the classical Fourier’s law. A significant characteristic of Pennes bioheat equation is that the velocity of heat diffusion is finite. Consequently, it has been used by many researchers who have improved mathematical models of heat transfer in organisms due to its simplicity. Shih et al. [33] used the Pennes bioheat model to investigate analytically the temperature response due to a sinusoidal heat flux on skin surface. Nevertheless, several studies proved that Pennes bioheat equation failed to elucidate the actual heat transport process between streams of blood flow in perfused biological tissues [34, 35]. Many computational and analytical techniques have been proposed to solve the biothermal transfer equation. Most of works have been analyzed numerically. Weinbaum et al. [35] and Nakayama et al. [36] proposed a number of bio-heat transfer equations for biological cells. The temperature profile in a malignant tumor cell treated by thermotherapy has been studied numerically by Khanafer et al. [37] using physiological velocity waveforms. Another technique to interpret the process of heat transport during hyperthermia is the variational iteration method (VIM). This technique was first introduced by He [38] and has been successful in solving the linear and nonlinear problems. The variable separation method was used by Kundu [39] to explain precisely the temperature change in biological tissues throughout therapeutic implementations. The thermal behavior in living tissues was investigated by Xu et al. [40, 41] using two different models, the Cattaneo-Vernotte (C-V) model with one phase lag time and the dual phase lag (DPL) model with two relaxation times. Kumar et al. [42] used the dual phase lag (DPL) method and finite element wavelet Galerkin method to investigate the biothermal variations during hyperthermia treatment. However, in these studies, it is assumed that the mechanical behavior has no impact on the temperature response. A thermal wave model has been employed by Liu et al. [43] to characterize the temperature distribution within living tissue. Ma et al. [44] used the (DPL) model with the usage of Green’s function method to study the thermo-mechanical response of human skin during heat therapy. In addition, Majchrzak and Stryczyski [45] examined the thermal transfer between arteries and living tissue using the DPL model. Liu et al. [46] examined multilayered skin temperature using the DPL bioheat transfer equation. Askarizadeh and Ahmadikia [47] studied burn durations in 2D skin tissue, employing DPL, thermal wave, and Pennes models. Shen et al. [48] proposed modified equations for heat-induced mechanical behavior in human skin. Iordana and Alexandru [49] suggested elastic parameters of tumor tissue affect magnetic nanoparticle distribution and temperature field.

Certainly, the thermal treatments such as hyperthermia look forward to a precise technique of thermoelastic behaviors in living cells for a better treatment influence. From literature, it has been shown that the bioheat equation serves as a fundamental tool for simulating heat distribution during hyperthermia treatment. However, a notable limitation in these studies is the lack of consideration for tissue elasticity, which can significantly influence the thermal response within the treated region. As such, it is essential to account for the thermoelastic behavior of tissues to obtain a more comprehensive and accurate representation of heat transfer during hyperthermia treatment. To the best of our knowledge, no previous studies were found that specifically investigate the thermoelastic response in biological tissue utilizing the generalized thermoelasticity theory. This indicates that there is a gap in the existing literature regarding the application of this theory to study the thermal responses in biological tissues. The objective of the present research is to effectively capture thermal responses over time and distance using the generalized thermoelasticity model. This method may be relatively close to real physics that takes into account the elasticity of biological tissues. The proposed model focuses on the examination of heat propagation within biological tissues, with the aim of understanding thermal responses triggered by laser irradiation. Its specific objective is to delve into the impact of heat arising from thermotherapy on a skin carcinoma. The effects of relaxation times on the temperature distribution at the tumor-normal tissue interface are calculated and visually represented through graphical presentations. Also, the impact of different laser irradiance on the thermal profile of the malignant tumor cells over a period of 2 minutes is interpreted graphically.

## 2. Mathematical formulations

During thermotherapy, nanoparticles that absorb the laser radiation are injected inside the tumor. Thus, nanoparticles exist plentifully inside cancer cells but not in adjacent normal tissues, so the applied laser directly heats the cancer. Tumors often exhibit inefficient lymphatic drainage, which means that once nanoparticles enter the tumor interstitium, they have a reduced ability to exit through the lymphatic system and they are unable to penetrate through tight endothelial junctions of normal blood vessels. This retention within the tumor microenvironment is part of the enhanced permeability and retention (EPR) effect, contributing to the selective accumulation of nanoparticles [50, 51]. In addition, the extracellular matrix (ECM) in tumor tissues can be altered compared to normal tissues. This altered ECM composition can facilitate the penetration and retention of nanoparticles within the tumor, contributing to their selective accumulation [52].

**Fig 1:**
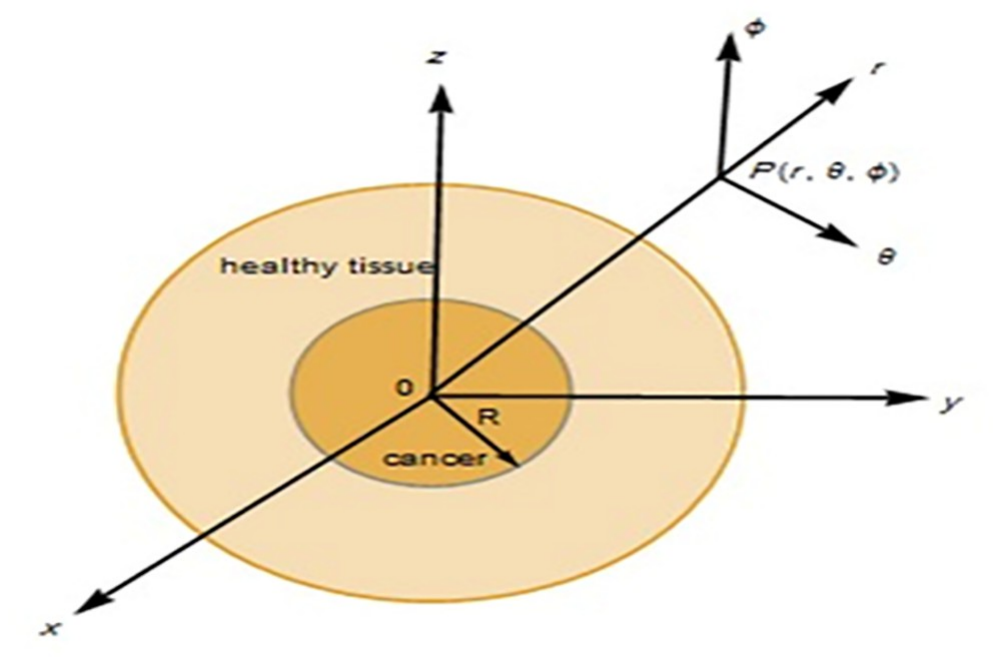
A spherical cancer with a radius R implanted in normal tissue.

The cancer is assumed to be a solid sphere with radius R inside the normal skin tissue as shown in Fig 1 [53].

The heterogeneous internal structure of the living tissue proposes the presence of a non-Fourier heat transfer behavior. Temperature variation in biological tissues was first noticed by Richardson et al.[54]. In the present study, the mathematical model used for thermal conduction in living tissues is the generalized thermoelasticity theory with one relaxation time [30, 55–57]. Laplace and inverse Laplace transforms are used to solve the problem. In this work, we consider a 1D problem. The field parameters of the sphere being considered exhibit axial symmetry, a characteristic arising from the symmetric shape of the sphere. In addition, the physical and thermal properties of the tumor tissue are assumed to be uniform along the radial direction. These assumptions lead to the simplification of the differential equation system into a 1D form (radial direction), that captures essential aspects of the biological thermal response during heat therapy on spheroidal skin tumor while minimizing computational complexity [53, 58]. The skin can be considered as a laminated composite structure, where each layer is assumed to be a homogeneous material with linear thermoelastic properties [59]. A linear thermoelastic model for analyzing the thermal response of skin tissue during hyperthermia cancer treatment is feasible, however it comes with important considerations and limitations. Linear thermoelasticity assumes a linear relationship between thermal expansion and temperature change. It is most accurate within a limited temperature range. If the hyperthermia treatment involves a moderate temperature increase within the linear regime like in our case, the model may provide reasonable approximations. For short hyperthermia treatments, where the thermal conditions do not lead to large and prolonged temperature changes, a linear thermoelastic model may be more applicable. However, for longer durations or extreme temperature changes, nonlinear effects may become more significant. As a result, a linear thermoelastic model can provide a computationally efficient approach for initial analyses and qualitative understanding of thermal response in skin tissues during hyperthermia treatment as it simplifies the analysis and reduces computational cost. In the future work, more sophisticated and possibly nonlinear models may be needed for a more realistic representation of the thermal behavior of skin tissues to address these limitations.

Cauchy’s equation of motion of elastic body is given by:

$$\sigma_{ij,j}+\rho F_{i}=\rho \frac{\partial^{2}u_{i}}{\partial t^{2}},$$ (1)

Where,

$$\sigma_{ij}=2\mu e_{ij}+\left[\lambda e-\gamma \left(T-T_{0}\right)\right]\delta_{ij},\;e=\frac{1}{2}\left(u_{i,j}+u_{j,i}\right).$$ (1*)

Where ***σ***_***ij***_, ***u***_***i***_ are the components of the stress and displacement respectively, ***F***_***i***_ is the external body force, *ρ* is the density of the medium and ***e*** is the cubical dilatation.

The total strain is given by

$$e_{ij}=e_{ij}^{\prime }+e_{ij}^{\prime \prime }.$$ (2)

Where $e_{ij}^{\prime }$ is the strain components due to free thermal expansion and $e_{ij}^{\prime \prime }$ is the elastic strain produced by the resistance of the medium to thermal expansion such that

$$e_{ij}^{\prime }=\alpha T_{i}\delta_{ij},\;e_{ij}^{\prime \prime }=\frac{1}{2\mu }\sigma_{ij}-\frac{\lambda \sigma_{ij}}{2\mu \left(3\lambda +2\mu \right)}\delta_{ij},$$ (3)

Where ***α*** is the coefficient of linear thermal expansion.

Substitute Eqs (1), (2) and (3) into Eq (1) we get Eq (4) which is the vector form of the equation of motion [60, 61]:

$$\mu_{i}\nabla^{2}u+\left(\lambda_{i}+\mu_{i}\right)grade_{i}-\gamma_{i}gradT_{i}=\rho_{i}\frac{\partial^{2}u}{\partial t^{2}},i=1,2.$$ (4)

The law of heat conduction formulated by Fourier is [41]:

$$q=-k_{i}\nabla T_{i},$$ (5)

Cattaneo extended Fourier’s law by adding the relaxation time *τ*_0_, this gives the modification of Fourier’s law as follows,

$$q+\tau_{0}\frac{\partial q}{\partial t}=-k_{i}\nabla T_{i},$$ (6)

Pennes equation is started in its most basic form as,

$$-\nabla .q=\rho c_{E}\frac{\partial T_{i}}{\partial t}-Q_{i}.$$ (7)

A modified Pennes bioheat transfer equation can be derived by combining Eq (6) with the Pennes Eq (7), which can be written as:

$$k_{i}\nabla^{2}T_{i}=\left(\frac{\partial }{\partial t}+\tau_{0}\frac{\partial^{2}}{\partial t^{2}}\right)\rho_{i}c_{Ei}T_{i}-\left(1+\tau_{0}\frac{\partial }{\partial t}\right)Q_{i}.$$ (8)

According to references [62–64] the metabolic heat generation rate and blood perfusion rates differ between tumor and normal tissue. However, in the study conducted by Maenosono and Saita [65], they considered the same values for metabolic heat generation and blood perfusion rates for both tumor and normal tissue. On the other hand, Andrä et al. [66] predicted the temperature distribution in breast tissue without accounting for the effects of blood perfusion and metabolism. The numerical simulations pertaining to the study were faced with challenges primarily due to uncertainties related to the impact of blood perfusion. To address this issue, the authors focused their considerations on small tumors situated in relatively homogeneous tissues with low blood perfusion as skin carcinomas. In skin carcinomas, the tumor is often situated in the superficial layers of the skin, which are primarily composed of fat and muscle tissues. These tissues may have relatively low metabolic heat generation and blood perfusion rates [67].

In our model we modified Eq (8) by adding the elastic term to apply the generalized thermoelasticity theory as follow:

$$k_{i}\nabla^{2}T_{i}=\left(\frac{\partial }{\partial t}+\tau_{0}\frac{\partial^{2}}{\partial t^{2}}\right)\left[\rho_{i}c_{Ei}T_{i}+\gamma_{i}T_{0}e_{i}\right]-\left(1+\tau_{0}\frac{\partial }{\partial t}\right)Q_{i}.$$ (9)

where *T*_*i*_ is the absolute temperature (*°C*), *Q*_*i*_ the strength of the heat source per unit volume. Regarding biological tissue, the heat source originates from *Q*_*b*_, *Q*_*m*_ and *Q*_*L*_ describe the perfusion heat source, the heat source of metabolic generation of tissue cell and heat generation due to nanoparticles respectively which has been widely used in many researches [46, 68–70]. The term *Q*_*b*_ = *w*_*bi*_*ρ*_*b*_*c*_*b*_ (*T*_*i*_ − *T*_0_) accounts for the heat exchange that occurs between the flowing blood and the tissue, where *w*_*bi*_ is the blood perfusion rates, *ρ*_*b*_ is the density of blood, *c*_*b*_ is the specific heat of the blood is neglected in the present study as its efficacy is stronger in larger tumors than for smaller tumors. In the case of smaller tumors where blood perfusion is relatively low, neglecting the perfusion heat source may not significantly impact the overall accuracy of the results. *Q*_*m*_ is not considered herein and *Q*_*L*_ = *αI*, *α* = *NC*_*abs*_ is the absorption coefficient of tissue (1 *cm*^−1^), N is the number of injected nanoparticles (number of NPs/ *cm*^3^), *C*_*abs*_ is the absorption cross-section area (*cm*^2^) and *I* is the laser irrandiance (wcm2) [71]. Therefore, the remaining volumetric heat source is *Q*_*i*_ = *Q*_*L*_ in the Eq (9) [72]. *ρ*_*i*_ is the density (*kgm*^−3^), *k*_*i*_ is the thermal conductivity (*Wm*^−1^°*C*^−1^), *τ*_0_ is the relaxation time (s), *c*_*Ei*_ is the specific heat in the absence of deformation (J/kgK), *λ*_*i*_ and *μ*_*i*_ are Lame’s constants, *γ*_*i*_ represents the thermal modulus whose relation with thermal expansion coefficient α_*ti*_ is *γ*_*i*_ = α_*ti*_ (2*μ*_*i*_ + 3*λ*_*i*_), *e*_*i*_ is the cubical dilatation, suffix *i* = 1,2 refers to the medium (*i* = 1 in the tumor and *i* = 2 in normal living tissue) and *T*_0_ represents the body temperature. If the influence of *e*_*i*_ on temperature is neglected, hence, the heat Eq (9) will be reduced to the conventional heat equation used in Pennes bioheat model.

The initial conditions are taken as:

ur,0=0∂u∂tr,0=0Tr,0=T0∂T∂tr,0=0. (9*)

*T*_0_ is the initial temperature such that $\frac{\left|\left.T-T_{0}\right|\right.}{T_{0}}\ll 1$, Hyperthermia treatment involves raising the tissue’s temperature above normal levels to selectively damage cancer cells while minimizing harm to healthy tissues.

In the context of studying the thermal response in cancerous skin tissue during hyperthermia treatment by using generalized thermoelasticity model, the condition "$\frac{\left|\left.T-T_{0}\right|\right.}{T_{0}}\ll 1$" is essential for analyzing the initial thermal response of the tissue during the treatment under the hyperthermia condition. Thus, provides insight into how the tissue responds to the thermal therapy.

Since,

$$\nabla^{2}u=grad\;div\;u\;–curl\;curl\;u,\;and\;div\;u=e.$$ (10)

Sub. From (10) into (8), we get

$$-\mu_{i\;}curl\;curl\;u+\left(\lambda_{i}+{2\mu }_{i}\right)grade_{i}\;-\gamma_{i}\;grad\;T_{i}=\rho_{i}\frac{\partial^{2}u}{\partial t^{2}}.$$ (11)

curl ***u*** must be equal zero because it gives the components of the vector *u* in the normal direction of it, since ***u*** = (*u*_*r*_, 0,0) that means there is no components in the direction of *θ and* ∅.

$$curl\;u=\frac{1}{r^{2}\;sin\theta }\left|\begin{array}{ccc} e_{r} & re_{\theta } & r\;sin\theta e_{\emptyset }\\ \frac{\partial }{\partial r} & \frac{\partial }{\partial \theta } & \frac{\partial }{\partial \emptyset }\\ u_{r} & 0 & 0 \end{array}\right|=\frac{1}{r^{2}\;sin\theta }\left[re_{\theta }\left(\frac{\partial u_{r}}{\partial \emptyset }\right)+rsin\theta \left(\frac{\partial u_{r}}{\partial \theta }\right)e_{\emptyset }\right],$$

but $\frac{\partial u_{r}}{\partial \emptyset }=\frac{\partial u_{r}}{\partial \theta }=0$, then, curl **u** = 0 ⟹ curl curl **u** = 0.

Then, Eq (11) becomes,

$$\left(\lambda_{i}+{2\mu }_{i}\right)grade_{i}\;-\gamma_{i}\;grad\;T_{i}=\rho_{i}\frac{\partial^{2}u}{\partial t^{2}},\;i=1,2$$ (12)

Also, the cubical dilatation *e*_*i*_ is thus given by

$$e_{i}=div\;u=\frac{1}{r^{2}}\frac{\partial }{\partial r}\left(r^{2}u_{r}\right),$$ (13)

Taking divergence of Eq (12), we get

$$\left(\lambda_{i}+{2\mu }_{i}\right)\left(\nabla^{2}-\rho_{i}\frac{\partial^{2}}{\partial t^{2}}\right)e_{i}\;-\gamma_{i}\nabla^{2}T_{i}=0.$$ (14)

The boundary conditions are taken as

T1R,t=T2R,tu1R,t=u2R,tqr1R,t=qr2R,tσrr1R,t=σrr2R,t. (15)

Where, *R* is the radius of the cancer cell, *q*_*r*1_ denotes the heat flux in the tumor and *q*_*r*2_ denotes the heat flux in the normal tissue.

## 3. Solution in the transformed domain

Applying Laplace transform which is defined by the formula [73]

$$\bar{f}\left(r,s\right)=\int_{0}^{\infty }f\left(r,t\right)e^{-st}dt.$$

Where, *f*(*r*,*t*) is an arbitrary function.

Appling Laplace transform for Eqs (9), (13) and (14), we get:

$$\left[k_{i}\nabla^{2}-\left(s+\tau_{0}s^{2}\right)\rho c_{E}\right]\bar{T_{i}}=\left(s+\tau_{0}s^{2}\right)\gamma_{i}T_{0}\bar{e_{i}}-\left(1+\tau_{0}s\right)\bar{Q_{i}},$$ (16)

$${\bar{e}}_{i}=\frac{1}{r^{2}}\frac{\partial }{\partial r}\left(r^{2}{\bar{u}}_{r}\right),$$ (17)

$$\left[\left(\lambda_{i}+{2\mu }_{i}\right)\nabla^{2}-\rho_{i}s^{2}\right]{\;\bar{e}}_{i}-\gamma_{i}grad{\bar{T}}_{i}=0.$$ (18)

From Eqs (12) and (14) by eliminating ${\bar{e}}_{i}$, we get

For Tumor

$$\left(\nabla^{4}-\left[\frac{\left(s+\tau_{0}s^{2}\right)\rho_{1}c_{E1}}{k_{1}}+\frac{k_{1}\rho_{1}s^{2}+\left(s+\tau_{0}s^{2}\right){\gamma_{1}}^{2}T_{0}}{k_{1}\left(\lambda_{1}+{2\mu }_{1}\right)}\right]\nabla^{2}+\frac{{\rho_{1}}^{2}s^{2}c_{E1}\left(s+\tau_{0}s^{2}\right)}{k_{1}\left(\lambda_{1}+{2\mu }_{1}\right)}\right){\bar{T}}_{1}=\frac{\left(\rho_{1}s^{2}\left(\lambda_{1}+{2\mu }_{1}\right)\nabla^{2}\right)\left(1+\tau_{0}s\right)}{k_{1}\left(\lambda_{1}+{2\mu }_{1}\right)}\bar{Q_{i}}.$$ (19)

For Tissue

$$\left(\nabla^{4}-\left[\frac{\left(s+\tau_{0}s^{2}\right)\rho_{2}c_{E2}}{k_{2}}+\frac{k_{2}\rho_{2}s^{2}+\left(s+\tau_{0}s^{2}\right){\gamma_{2}}^{2}T_{0}}{k_{2}\left(\lambda_{2}+{2\mu }_{2}\right)}\right]\nabla^{2}+\frac{{\rho_{2}}^{2}s^{2}c_{E2}\left(s+\tau_{0}s^{2}\right)}{k_{2}\left(\lambda_{2}+{2\mu }_{2}\right)}\right){\bar{T}}_{2}=0.$$ (20)

The solution of Eq (19), is given by:

$${\bar{T}}_{1}=\frac{A_{1}}{\sqrt{r}}I_{\frac{1}{2}}\left(m_{1}r\right)+\frac{A_{2}}{\sqrt{r}}I_{\frac{1}{2}}\left(m_{2}r\right)+\frac{q_{1}}{\rho_{1}{s^{2}c}_{E1}},$$ (21)

where *q*_1_ is a constant

Also, for Eq (20)

$${\bar{T}}_{2}=\frac{B_{1}}{\sqrt{r}}K_{\frac{1}{2}}\left(n_{1}r\right)+\frac{B_{2}}{\sqrt{r}}K_{\frac{1}{2}}\left(n_{2}r\right),$$ (22)

where $I_{\frac{1}{2}}\left(m_{i}r\right)$ and $K_{\frac{1}{2}}\left(n_{i}r\right)$ are the modified Bessel of the second kind of order $\frac{1}{2},\;i=1,2$.

Using Eqs (13), (14) with (17) and (18), we get:

For tumor

$${\bar{e}}_{1}=\frac{\gamma_{1}{m_{1}}^{2}A_{1}}{\left(\lambda_{1}+{2\mu }_{1}\right){m_{1}}^{2}-\rho_{1}s^{2}}.\frac{I_{\frac{1}{2}}\left(m_{1}r\right)}{\sqrt{r}}+\frac{\gamma_{2}{m_{2}}^{2}A_{2}}{\left(\lambda_{1}+{2\mu }_{1}\right){m_{2}}^{2}-\rho_{1}s^{2}}.\frac{I_{\frac{1}{2}}\left(m_{2}r\right)}{\sqrt{r}}.$$ (23)

$${\bar{u}}_{1}=\frac{\gamma_{1}m_{1}A_{1}}{\left(\lambda_{1}+{2\mu }_{1}\right){m_{1}}^{2}-\rho_{1}s^{2}}.\frac{I_{\frac{3}{2}}\left(m_{1}r\right)}{\sqrt{r}}+\frac{\gamma_{2}m_{2}A_{2}}{\left(\lambda_{1}+{2\mu }_{1}\right){m_{2}}^{2}-\rho_{1}s^{2}}.\frac{I_{\frac{3}{2}}\left(m_{2}r\right)}{\sqrt{r}}.$$ (24)

For tissue

$${\bar{e}}_{2}=\frac{\gamma_{2}{n_{1}}^{2}B_{1}}{\left(\lambda_{2}+{2\mu }_{2}\right){n_{1}}^{2}-\rho_{2}s^{2}}.\frac{K_{\frac{1}{2}}\left(n_{1}r\right)}{\sqrt{r}}+\frac{\gamma_{2}{n_{2}}^{2}B_{2}}{\left(\lambda_{2}+{2\mu }_{2}\right){n_{2}}^{2}-\rho_{2}s^{2}}.\frac{K_{\frac{1}{2}}\left(n_{2}r\right)}{\sqrt{r}}.$$ (25)

$${\bar{u}}_{2}=\frac{-\gamma_{2}n_{1}B_{1}}{\left(\lambda_{2}+{2\mu }_{2}\right){n_{1}}^{2}-\rho_{2}s^{2}}.\frac{K_{\frac{3}{2}}\left(n_{1}r\right)}{\sqrt{r}}-\frac{\gamma_{2}n_{2}B_{2}}{\left(\lambda_{2}+{2\mu }_{2}\right){n_{2}}^{2}-\rho_{2}s^{2}}.\frac{K_{\frac{3}{2}}\left(n_{2}r\right)}{\sqrt{r}}.$$ (26)

The Heat flux is given by the following equation:

$$q_{ri}=\frac{-k_{i}}{1+\tau_{0}s}\frac{\partial {\bar{T}}_{i}}{\partial r}.$$ (27)

For tumor

$$q_{r1}=\frac{-k_{1}}{1+\tau_{0}s}\frac{\partial {\bar{T}}_{1}}{\partial r}.$$

$${\bar{q}}_{r1}=\frac{-k_{1}}{1+\tau_{0}s}\left(\frac{A_{1}}{\sqrt{r}}m_{1}I_{\frac{3}{2}}\left(m_{1}r\right)+\frac{A_{2}}{\sqrt{r}}{m_{2}I}_{\frac{3}{2}}\left(m_{2}r\right)\right).$$ (28)

For tissue

$$q_{r2}=\frac{-k_{2}}{1+\tau_{0}s}\frac{\partial {\bar{T}}_{2}}{\partial r}.$$

$${\bar{q}}_{r2}=\frac{-k_{2}}{1+\tau_{0}s}\left(\frac{B_{1}}{\sqrt{r}}n_{1}K_{\frac{3}{2}}\left(n_{1}r\right)+\frac{B_{2}}{\sqrt{r}}{n_{2}K}_{\frac{3}{2}}\left(n_{2}r\right)\right).$$ (29)

We have for the tumor

$${\bar{\sigma }}_{rr1}=\lambda_{1}{\bar{e}}_{1}+2\mu_{1}\frac{\partial {\bar{u}}_{1}}{\partial r}-\gamma_{1}\left({\bar{T}}_{1}-\frac{T_{0}}{s}\right),$$ (30)

i.e.


$$\begin{array}{ll} {\bar{\sigma }}_{rr1}\;= & A_{1}\left[\frac{\gamma_{1}{m_{1}}^{2}\left(\lambda_{1}+2\mu_{1}\right)}{\left(\lambda_{1}+2\mu_{1}\right){m_{1}}^{2}-\rho_{1}s^{2}}.\frac{I_{\frac{1}{2}}\left(m_{1}r\right)}{\sqrt{r}}-\gamma_{1}.\frac{I_{\frac{1}{2}}\left(m_{1}r\right)}{\sqrt{r}}-\frac{4\mu_{1}\gamma_{1}m_{1}}{\left(\lambda_{1}+2\mu_{1}\right){m_{1}}^{2}-\rho_{1}s^{2}}.\frac{I_{\frac{3}{2}}\left(m_{1}r\right)}{\sqrt{r^{3}}}\right]+\\ & A_{2}\left[\frac{\gamma_{1}{m_{2}}^{2}\left(\lambda_{1}+2\mu_{1}\right)}{\left(\lambda_{1}+2\mu_{1}\right){m_{2}}^{2}-\rho_{1}s^{2}}.\frac{I_{\frac{1}{2}}\left(m_{2}r\right)}{\sqrt{r}}-\gamma_{1}.\frac{I_{\frac{1}{2}}\left(m_{2}r\right)}{\sqrt{r}}-\frac{4\mu_{1}\gamma_{1}m_{2}}{\left(\lambda_{1}+2\mu_{1}\right){m_{2}}^{2}-\rho_{1}s^{2}}.\frac{I_{\frac{3}{2}}\left(m_{2}r\right)}{\sqrt{r^{3}}}\right]-\\ & \frac{\gamma_{1}q_{1}}{\rho_{1}s^{2}c_{E1}}+\frac{\gamma_{1}T_{0}}{s}. \end{array}$$ (31)

For tissue

$$\begin{array}{ll} {\bar{\sigma }}_{rr2}\;= & B_{1}\left[\frac{\gamma_{2}{n_{1}}^{2}\left(\lambda_{2}+2\mu_{2}\right)}{\left(\lambda_{2}+2\mu_{2}\right){n_{1}}^{2}-\rho_{2}s^{2}}.\frac{K_{\frac{1}{2}}\left(n_{1}r\right)}{\sqrt{r}}-\gamma_{2}.\frac{K_{\frac{1}{2}}\left(n_{1}r\right)}{\sqrt{r}}+\frac{4\mu_{2}\gamma_{2}n_{1}}{\left(\lambda_{2}+2\mu_{2}\right){n_{1}}^{2}-\rho_{2}s^{2}}.\frac{K_{\frac{3}{2}}\left(n_{1}r\right)}{\sqrt{r^{3}}}\right]+\\ & B_{2}\left[\frac{\gamma_{2}{n_{2}}^{2}\left(\lambda_{2}+2\mu_{2}\right)}{\left(\lambda_{2}+2\mu_{2}\right){n_{2}}^{2}-\rho_{2}s^{2}}.\frac{K_{\frac{1}{2}}\left(n_{2}r\right)}{\sqrt{r}}-\gamma_{2}.\frac{K_{\frac{1}{2}}\left(n_{2}r\right)}{\sqrt{r}}+\frac{4\mu_{2}\gamma_{2}n_{2}}{\left(\lambda_{2}+2\mu_{2}\right){n_{2}}^{2}-\rho_{2}s^{2}}.\frac{K_{\frac{3}{2}}\left(n_{2}r\right)}{\sqrt{r^{3}}}\right]+\\ & \frac{\gamma_{2}T_{0}}{s}. \end{array}$$ (32)

Applying the boundary conditions (15) on the Eqs (21), (22), (24), (26), (28), (29), (31) and (32) we get the constants *A*_1_, *A*_2_, *B*_1_, *B*_2_ the we get the complete solution of our problem.

## 4. Results and discussion

In this manuscript, the authors introduce a novel approach to studying heat therapy for cancerous cells within human tissue, specifically using the generalized theory of thermoelasticity. Previous researches in this area have primarily relied on the heat equation only [12, 23, 37, 40, 42], assuming the human body behaves like a solid material. However, the authors propose a different perspective by considering the human skin as an elastic material, which better represents the physical reality. We assume that a spherical cancer tissue of radius 0.02 m [53] is imbedded in healthy skin tissue. To investigate the heat transmission in both the healthy and tumor cells within the cutaneous areas of the human skin, the authors employ the generalized thermoelasticity model. The thermophysical properties of human skin and blood that were used in the numerical computations are listed in Table 1 [53]. The initial temperature is set at T_0_ = 37°*c* and the thermal relaxation time *τ*_0_ = 2 s.

**Table 1:** Thermophysical properties.

Property	Value	Property	Value
Tumor tissue with AuNPs		**Normal tissue**	
**Density ρ_1_ (kg/m^3^)**	1660	Density ρ_2_ (kg/m^3^)	1000
**Specific heat capacity C_1_ (J/kgK)**	2540	Specific heat capacity C_2_ (J/kgK)	3720
**Thermal conductivity K_1_ (W/mK)**	0.778	Thermal conductivity K_2_ (W/mK)	0.642
**Lame constant μ_1_ (kg/ms^2^)**	0.4	Lame constant *μ*_2_ (*kg*/*ms*^2^)	0.4
**Lame constant λ_1_ (kg/ms^2^)**	28571	Lame constant *λ*_2_ (*kg*/(*ms*^2^)	10000
**Linear thermal expansion E_1_ (KPa)**	15	Linear thermal expansion *E*_2_ (KPa)	5
**Relaxation time (s)**	2	Relaxation time (s)	2

The duplicated and uncontrollable growth of cancerous cells leads to anomalous temperature variations in the adjacent healthy tissues. Thermal therapy such as hyperthermia treatment, includes transmitting a focused powerful laser irradiation to the tumor’s source, which can destroy the malignant tissues. However, the persistent use of heat may hurt the healthy cells that surround the cancer. It is therefore crucial to examine the temperature distribution in both healthy and tumor cells. A mathematical formula based on the generalized thermoelasticity theory was used to assess the alteration in the cell temperature during hyperthermia, providing a more accurate representation of heat behavior in this biological system. By adopting this model, the study aims to gain deeper insights into the dynamics of heat distribution and its effects on the tumor and surrounding healthy tissue during heat therapy.

Biological tissues have physical properties that vary between cancerous tissues and healthy tissues. This abrupt difference may have a significant impact on the temperature elevation during thermal therapy. The temperature variation in the malignant tissues (0 ≤ r ≤ R) and in healthy tissues (R ≤ r ≤ ∞) relies on the time, t and the distance, r, from the center of the cancer. This dependence on time and distance stems from several factors: 1) thermophysical properties: Cancerous tissues often have different thermophysical properties compared to healthy tissues. These properties include thermal conductivity, specific heat, and density, which determine how tissues conduct and store heat. The differences in these properties contribute to variations in temperature within the tissue. 2) blood perfusion: Blood flow in tissues affects their ability to dissipate heat. Malignant tissues can exhibit altered vasculature, which may lead to reduced blood perfusion compared to healthy tissues. This reduced blood flow can result in lower heat dissipation, leading to higher temperatures in the cancerous region. 3) the tumor microenvironment, characterized by factors like extracellular matrix composition and immune cell infiltration, can also influence temperature variations within the cancerous tissues. 4) heat source: The external heat source applied during thermal therapy, such as microwaves, ultrasound, or focused laser, will also impact the temperature distribution. The location, intensity, and duration of the heat source will determine how heat is distributed within the tissues [74, 75]. Understanding the interplay between these factors is critical for optimizing thermal therapy for skin cancer and other medical applications. The proposed mathematical model can help simulate and predict temperature distributions accurately, considering the complex nature of tissue interactions.

**Fig 2:**
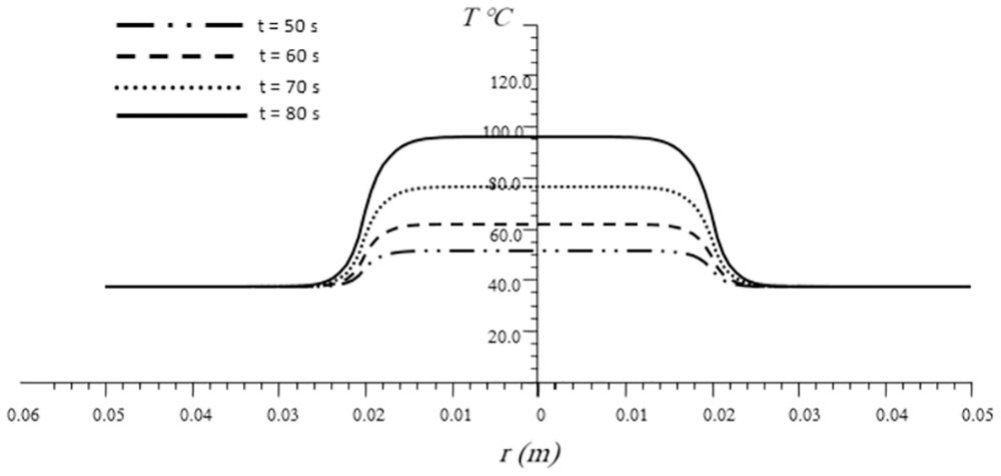
Impact of different time instance t on the temperature distribution versus the radial position in the cancer and the healthy tissues.

Fig 2 represents the temperature difference in cancerous and healthy cells for different time instances (t = 50, 60, 70 and 80 seconds) across the radial distance (r). As shown, it was found that the time instance parameter has a significant impact on the temperature distribution in the cancer and normal cells. The figure demonstrates that with the increase in time, the thermal temperature increases. On the other hand, with the increase of radial distance r in the direction of heat transfer, the temperature decreased for all time instance. This result revealed that the temperature propagation inside the medium moves slowly, which is in agreement with physics. This situation also emphasizes the significance of the generalized thermoelastic model as opposed to the classical model (coupled and uncoupled) that predicts infinite speed. It was noted that at high time instances (70 and 80 seconds), the temperature reaches high levels that’s because the generalized thermoelasticity theory turned to coupled theory according to reference [76] which confirmed the validity of our proposed model that gives reasonable, realistic results at low time instances. In addition, at high time instances, the temperature rises to high levels as previous studies based on PBT equations. The results emphasize the superiority of the generalized thermoelastic model in accurately describing the thermal behavior within the biological medium during thermal therapy for skin cancer. The current model can be used to explain various thermotherapeutic methods. It can have a profound effect on enhancing clinical results. This is due to the fact that the extent of thermal damage is influenced by both the temperature of the tissue and the duration of exposure.

In the field of applied biological sciences focusing on living tissue, the precise identification of burns stands out as a crucial aspect, constituting an essential component of thermal therapy. Moritz and Henriques [77, 78] introduced a relationship for assessing thermal injuries, providing a means to quantify thermal damage using the non-dimensional parameter Ω based on the distribution of skin temperature:

$$\bar{\Omega }\;\left(r,s\right)=\int_{0}^{t}Be^{\left(\frac{E_{a}}{R_{g}}\right)/\bar{T}(r,s)}dt.$$ (33)

Where *B* is the factor of tissue frequency, *B* = 3.1*10^98^ *s*^−1^, *E*_*a*_ describes the activation energy, *E*_*a*_ = 6.28 * 10^5^ J/mol, and *R*_*g*_ represents the universal gas constant *R*_*g*_ = 8.313 J/molK.

Table 2 represents the thermal damage that occurs on the skin over time. The numerical findings indicated that the thermal damage to normal tissue escalated with time. Furthermore, it is evident that the thermal damage values approach zero, which means minimal thermal damage occurs to normal tissue during hyperthermia treatment in such instances.

**Table 2:** Thermal damage on normal tissue due to hyperthermia therapy over time.

Time (s)	Thermal Damage of Normal Tissue
***t* = 50**	2.42 * 10^−10^
***t* = 60**	7.724 * 10 ^**−8**^
***t* = 70**	6.031 * 10^−5^
***t* = 80**	1.166 * 10 ^**−4**^

**Fig 3:**
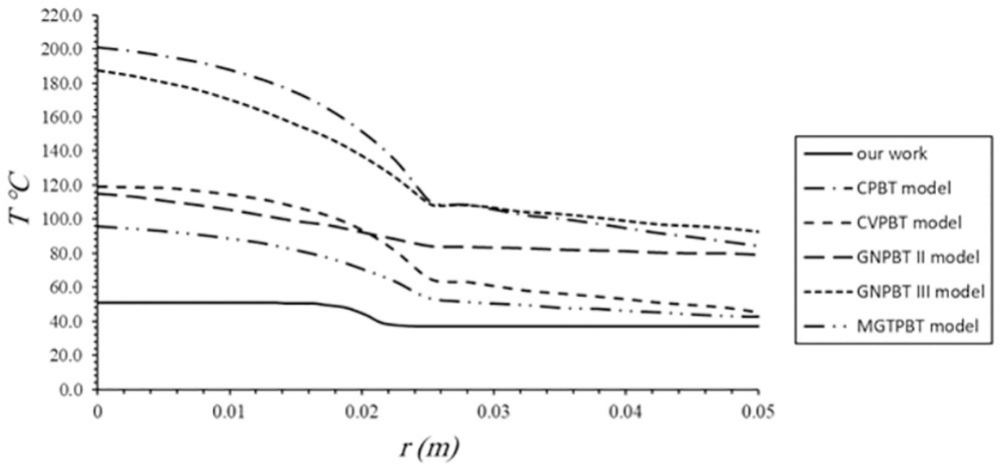
Temperature distribution in the skin carcinoma and adjacent healthy tissues for various models based on the PBTequation.

Fig 3 shows a comparison of our obtained outcomes regarding temperature distributions within skin carcinoma and the adjoining healthy tissues versus the radial distance with various models based on the PBT equation at t = 50 s. Our findings exhibit that during hyperthermia treatment, the temperature rises solely within the tumor, while in the neighboring healthy tissues, it declines immediately beyond the target zone, returning to the standard body temperature of 37°C. This leads to damage and killing the cancerous cells while protecting the surrounding healthy tissues. Although, all the models based on PBT equation include the blood perfusion rate which functions as a coolant and prevent tissue temperature elevation, all these models have exhibited the greatest temperature levels within and around the tumor. It’s noteworthy that this decline is merely a characteristic behavior; in terms of magnitude, temperatures remain notably elevated both inside and outside the tumor, and none of these models return the temperature to its normal level outside the cancer in the adjacent normal tissues. Among these models, the conventional Pennes bioheat model (CPBT) provides the highest temperature. Including the cubical dilatation e, in our model, leads to temperature escalation within the tumor beyond cytotoxic thresholds, while simultaneously avoiding excessive exposure of normal tissues. Although the graphical representation shows quite resemblances in behavior between the Moore—Gibson—Thompson Pennes bioheat transfer model (MGTPBT) and our proposed model, the magnitude of temperature elevation is greater in the MGTPBT model compared to ours. Additionally, the Cattaneo-Vernotte Pennes bioheat model (CVPBT) exhibits behavior similar to the MGTPBT model, with slight discrepancies in temperature magnitude. Noteworthy differences emerge in thermal response between Green and Naghdi’s theory of type II (GNPBT II) and type III (GNPBT III), attributed to energy losses in the first case. Furthermore, the GNPBTIII model converged with CPBT model, in contrast to all Pennes-modified biothermal models. The results presented in this study could hold great significance for biomedical thermal therapy, which involves both normal and diseased cells. In addition, these results contribute to the development of theoretical understanding in the field of vital heat transfer within the spherical tissue structure.

**Fig 4:**
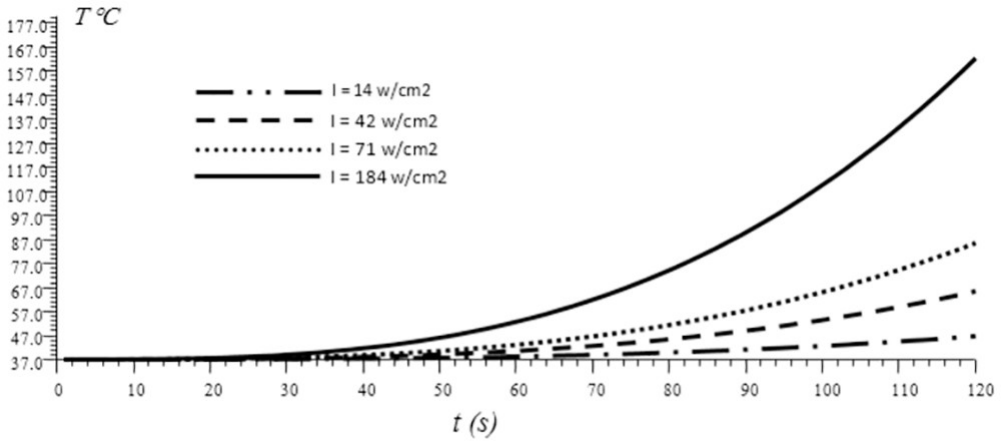
Temperature rise of a cancer cell in skin tissue over a period of 2 minutes at different laser irradiance.

Fig 4 shows the numerical results obtained from non-Fourier model indicating the effect of laser irradiance on the temperature variation during two minutes of thermotherapy. It was obviously shown that at low laser irradiance, the temperature elevated monotonically but slowly over time. While in higher laser irradiation, the temperature rose continuously with a fast rate throughout the treatment period. This observation emphasizes the significant influence of laser irradiance on the temperature dynamics during thermotherapy. The laser’s irradiance or energy input directly affects how rapidly the tissue heats up, with higher irradiance leading to quicker and more pronounced temperature elevations. Careful control of laser parameters is crucial to ensure effective treatment outcomes and minimize any potential adverse effects on the surrounding healthy tissues.

**Fig 5:**
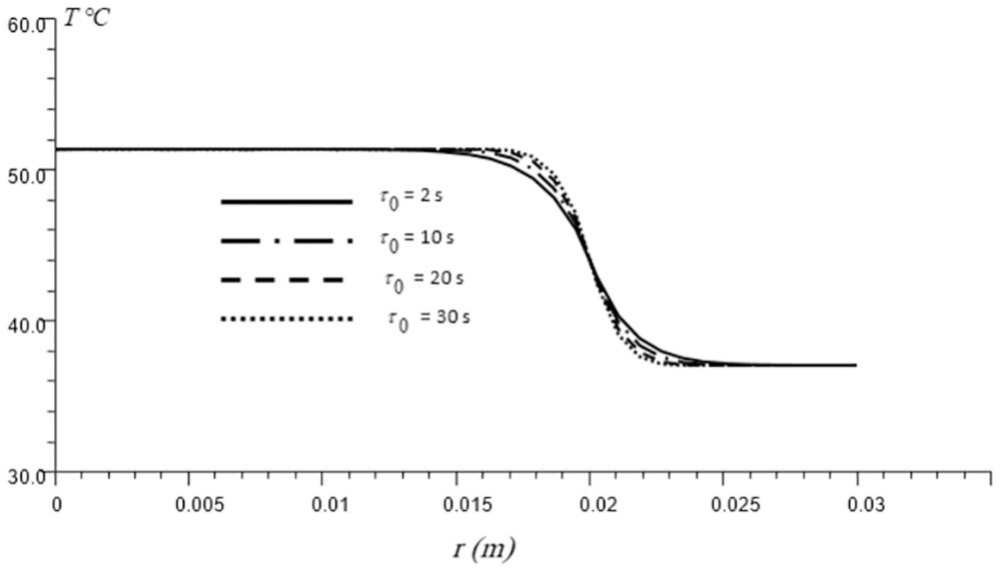
Temperature distribution in generalized thermoelasticity theory under different relaxation times. The time is t = 50 s.

The relaxation time value for homogeneous substances can be computed theoretically [79, 80], which is not convenient for materials with heterogeneous internal Structures as skin. The majority of biological substances are heterogeneous, and thus their heat relaxation times are much greater when compared to engineering substances. For homogeneous materials, the value of the relaxation time given by is in the range 10^−8^ − 10^−14^ s. A study examined by Vedavarz et al. [81] revealed that the thermal relaxation time of biological tissues has a value in the range of 1–100 s. In addition, Luikov [82] found that the value of the relaxation time for non-homogeneous materials is in the range 10^−3^ − 10^3^ s. However, no information on the thermal relaxation time of the skin tissue has been reported. Even for living cells, there are few experimental studies published [40]. According to relaxation theory [83], there is a strong correlation that the relaxation time of most living tissues shows a marked dependence on temperature. The exact knowledge of the alteration in temperature profile inside the living tissues is significant when translating results from analytical analysis to in vivo such as the case in thermotherapy studies. Fig 5 depicts the temperature profile with several relaxation times. As shown from Fig 5, the change in the thermal relaxation time can slightly increase the heat diffusion velocity. It is evident that by increasing the relaxation time, the temperature near the thermal source is lower while the temperature is higher further away from this region. In addition, the effect of changing the relaxation time appears strongly around the surface of the embedded tumor in skin tissue.

Due to the high sensitivity of living cells to changes in temperature over space and time, it is crucial to precisely examine the thermal response caused by the subjected laser irradiance during the hyperthermia process to ensure the therapeutic effectiveness by destroying the malignant tumor cells and minimize the side effects.

## 5. Conclusions

In this research, an investigation into the thermoelastic responses of skin tissue containing an embedded spheroidal tumor when exposed to a thermal shock load is conducted. The study was based on the framework of generalized thermoelasticity theory with one relaxation time as it has the potential to describe the interaction between the microstructural elements of matter during the thermo-therapeutic process. The model presented in this study offers a novel and insightful perspective on the propagation of thermal waves. It represents the first attempt to explore thermoelastic behavior in biological tissues using the generalized thermoelasticity approach. It has the potential to provide deeper insights into the heat transfer mechanisms and temperature variations within the skin. Ultimately, this contributes to optimizing thermal treatments, enhancing their efficiency, and ensuring safety.

- A comparison between the present generalized thermoelasticity model and different models based on PBT regarding temperature distributions within skin carcinoma and the adjoining healthy tissues has been conducted. This comparative analysis underscores that our proposed model yields more realistic, accurate and dependable predictions when contrasted with previously employed models. The outcomes shed light on the impact of spatiotemporal temperature distributions on skin tissue and that the selection of a specific model for the heat conduction equation has a substantial impact on how biological tissues response to the thermotherapy.
- It was found that the selection of the appropriate laser irradiance is essential for optimizing thermotherapy procedures. This selection significantly impacts the attainment of the desired temperature distribution and therapeutic outcomes while considering safety concerns.
- It was found that the relaxation time used has a significant influence on the temperature distribution in the tumor and the normal tissues. It is observed that by decreasing the thermal relaxation time, the heat transfer capacity of the diseased medium can be increased. Whereas, the presence of thermal relaxation coefficient reduced the temperature drop. Therefore, in low relaxation time, the temperature drop is small which will in turn minimize the side effect of the therapy on the skin.
- The findings and results obtained from this model have significantly contributed to our understanding of how biological tissues respond to thermal stimuli. The current model can be used to explain various thermotherapeutic methods. It can have a profound effect on enhancing clinical results. This is due to the fact that the extent of thermal damage is influenced by both the temperature of the tissue and the duration of exposure. Thus, our model reaches the goal of hyperthermia treatment in a short duration taking into account the elasticity of the biological tissues to avoid the thermal damage.
- More detailed experimental studies are necessary to measure the exact value of the thermal relaxation time during the heat transfer process in living tissues. Other types of tumors located in more complex and vascularized organs and large tumor size are another items of further investigations taking into account the influence of blood perfusion in order to improve the effectiveness of cancer treatment strategies.
